# PIM Kinase Inhibition Attenuates the Malignant Progression of Metastatic Hepatoblastoma

**DOI:** 10.3390/ijms25010427

**Published:** 2023-12-28

**Authors:** Janet R. Julson, Colin H. Quinn, Swatika Butey, Michael H. Erwin, Raoud Marayati, Nazia Nazam, Jerry E. Stewart, Elizabeth A. Beierle

**Affiliations:** Division of Pediatric Surgery, Department of Surgery, University of Alabama at Birmingham, 1600 7th Ave. South, Lowder Building, Suite 300, Birmingham, AL 35233, USA; jjulson@uabmc.edu (J.R.J.);

**Keywords:** hepatoblastoma, PIM kinase, small molecule inhibition, ataxia telangiectasia mutated (ATM)

## Abstract

Hepatoblastoma is the most common primary pediatric liver tumor. Children with pulmonary metastases at diagnosis experience survival rates as low as 25%. We have shown PIM kinases play a role in hepatoblastoma tumorigenesis. In this study, we assessed the role of PIM kinases in metastatic hepatoblastoma. We employed the metastatic hepatoblastoma cell line, HLM_2. PIM kinase inhibition was attained using PIM3 siRNA and the pan-PIM inhibitor, AZD1208. Effects of PIM inhibition on proliferation were evaluated via growth curve. Flow cytometry determined changes in cell cycle. AlamarBlue assay assessed effects of PIM kinase inhibition and cisplatin treatment on viability. The lethal dose 50% (LD_50_) of each drug and combination indices (CI) were calculated and isobolograms constructed to determine synergy. PIM kinase inhibition resulted in decreased HLM_2 proliferation, likely through cell cycle arrest mediated by p21. Combination therapy with AZD1208 and cisplatin resulted in synergy, potentially through downregulation of the ataxia-telangiectasia mutated (ATM) kinase DNA damage response pathway. When assessing the combined effects of pharmacologic PIM kinase inhibition with cisplatin on HLM_2 cells, we found the agents to be synergistic, potentially through inhibition of the ATM pathway. These findings support further exploration of PIM kinase inhibition as a therapeutic strategy for metastatic hepatoblastoma.

## 1. Introduction

Hepatoblastoma remains the most common primary liver malignancy in children. In the United States, the annual incidence is approximately 2 per 1,000,000 children [1]. Compared to other pediatric cancer types, diagnoses of hepatoblastoma have seen the most dramatic increase in incidence over the past several decades [2]. One of the most important prognostic factors is the presence of pulmonary metastatic disease at diagnosis, which may occur in up to 20% of patients. For these children, event free survival is as low as 25% [3].

Despite the increase in incidence, the treatment strategy for hepatoblastoma has remained largely unchanged for the last two decades, consisting of complete tumor resection for cure, but relying on chemotherapeutics for patients whose tumors are unresectable upfront and to reduce the rates of postoperative recurrence. Cisplatin, a DNA damage inducing agent, remains the mainstay of therapy, but is associated with chemoresistance as well as serious long-term toxicities including neuro- and nephrotoxicity among others with life-long implications [4,5].

The lack of hepatoblastoma cell lines and paucity of targetable mutations has hampered progress in developing new therapies but has opened the door to evaluate the effects of kinase inhibitors [6,7]. One targetable family of kinases includes the serine/threonine PIM kinases. This family consists of PIM1, PIM2, and PIM3 which have been shown to play roles in tumorigenesis in several cancer types. In primary hepatoblastoma, PIM3 correlates with worse patient prognosis, increased tumor cell proliferation, motility, stemness, and drug resistance [8]. Because metastatic tumors cells behave differently than primary tumors cells, we previously established a metastatic hepatoblastoma cell line to better study this specific disease process [9]. In the following study, we explore the effects of PIM kinase inhibition in metastatic hepatoblastoma.

## 2. Results

### 2.1. Metastatic Hepatoblastoma Cells Express PIM Kinases

PIM kinases have been demonstrated to play a role in a number of pediatric solid tumors and have been shown to be of particular importance in hepatoblastoma [8,9,10]. We first used immunoblotting to confirm the expression of each of the individual PIM kinases in the metastatic hepatoblastoma cell line (Figure S1A), providing evidence for the presence of a druggable target for the pan-PIM inhibitor, AZD1208, which was employed in the remainder of our studies.

### 2.2. PIM Kinase Inhibition Results in Decreased Metastatic Hepatoblastoma Cell Proliferation and Impedes Progression through the Cell Cycle

**Figure 1:**
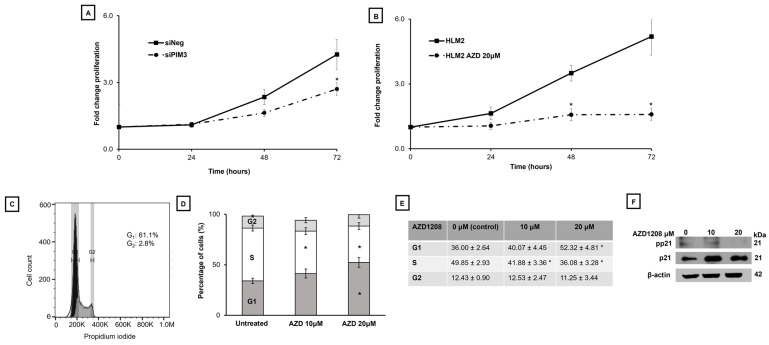
PIM kinase inhibition results in decreased metastatic hepatoblastoma cell proliferation and cell cycle progression. (**A**) After siRNA knockdown of PIM3, 5 × 10^5^ HLM_2 cells were plated and counted over the course of 72 h. There was a significant decrease in HLM_2 proliferation with decreased PIM3 expression compared to control at 72 h (4.2 ± 0.7 v. 1.2 ± 0.3, *p* ≤ 0.05). (**B**) HLM_2 cells (5 × 10^4^ cells per well) were plated in 12-well plates, allowed to adhere, and treated with AZD1208 (0, 20 µM) for 24, 48, or 72 h. There was a significant decrease in proliferation at 48 h (3.5 ± 0.4 v. 1.6 ± 0.3, *p* ≤ 0.05) and at 72 h (5.2 ± 0.9 v. 1.6 ± 0.3, *p* ≤ 0.05) after PIM kinase inhibition. (**C**) HLM_2 cells (1 × 10^6^) were plated in low serum (4% FBS) media, allowed to attach, and incubated for 24 h with AZD1208 (0, 10, 20 µM). Flow cytometry was utilized to assess the effects of PIM kinase inhibition on the cell cycle. A representative histogram is presented. (**D**) Graphic and (**E**) tabular representation of the results (mean percent cells in phase ± SEM) of the cell cycle analysis from three biologic replicates are shown. Treatment with AZD1208 decreased progression of G_1_ to S phase as demonstrated by the decrease in the percentage of cells in the S phase (49.85 ± 2.93 v. 36.08 ± 2.47, control v. AZD1208 20 µM, *p* ≤ 0.05) and a statistically significant increase in the percentage of cells in G_1_ (36.00 ± 2.64 v. 52.32 ± 4.81, control v. AZD1208 20 µM, *p* ≤ 0.05) after treatment. (**F**) Immunoblotting demonstrates a decrease in the phosphorylation of p21 after treatment with AZD1208. Total p21 expression remained stable. β-actin served as a loading control. Data are reported as mean ± standard error of the mean (SEM) and represent at least three biologic replicates. * *p* ≤ 0.05.

We began by exploring the effects of PIM kinase inhibition on metastatic hepatoblastoma cell proliferation. Prior studies in our lab have shown PIM3 to be of particular importance in hepatoblastoma [8,10] thus we began by evaluating the effects of PIM3 knockdown on HLM_2 cell proliferation. Immunoblotting confirmed knockdown of PIM3 (Supplemental Figure S1B). We found a statistically significant decrease in HLM_2 cell proliferation at 72 h after knockdown of PIM3 (4.2 ± 0.7 v. 1.2 ± 0.3, siNeg v. siPIM3, *p* ≤ 0.05) (Figure 1A). After employing pan-PIM kinase inhibition with AZD1208 (20 µM), we found a significant decrease in proliferation at both 48 h (3.5 ± 0.4 v. 1.6 ± 0.3, control v. AZD1208, *p* ≤ 0.05) and at 72 h (5.2 ± 0.9 v. 1.6 ± 0.3, control v. AZD1208, *p* ≤ 0.05) (Figure 1B).

To explore the mechanism underpinning this change in proliferation, we evaluated the effects of PIM kinase inhibition on cell cycle. AZD1208 treatment decreased progression from G_1_ to S phase. There was a decrease in percentage of cells in S phase after treatment with AZD1208 at 10 µM (49.85 ± 2.93 v. 41.88 ± 3.36%, control v. AZD1208, *p* ≤ 0.05) and 20 µM (49.85 ± 2.93 v. 36.08 ± 2.47%, control v. AZD1208, *p* ≤ 0.05). The percentage of cells in G_1_ increased with AZD1208 (20 µM) treatment (52.32 ± 4.81 v. 36.00 ± 2.64%, AZD1208 v. control, *p* ≤ 0.05) (Figure 1C–E). Immunoblotting demonstrated that the change in cell cycle progression was likely mediated through decrease in phosphorylation of p21 (CDKN1A), a known modulator of the cell cycle at G_1_ [11] (Figure 1F).

### 2.3. PIM Kinase Inhibition Decreased Metastatic Hepatoblastoma Cell Stemness

**Figure 2:**
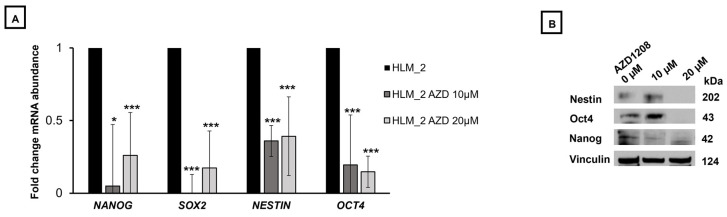
PIM kinase inhibition decreased metastatic hepatoblastoma cell stemness markers. (**A**) HLM_2 (1 × 10^6^) cells were treated with increasing concentrations of AZD1208 (0, 10, or 20 µM) for 72 h. The RNA was collected and qPCR completed. Treatment with AZD1208 (10 µM) decreased the mRNA abundance of each of the markers; *Nanog* (1.00 ± 0.0 v. 0.45 ± 0.47, control v. AZD1208 10 µM, *p* ≤ 0.05), *SOX2* (1.00 ± 0.0 v. 0.25 ± 0.34, control v. AZD1208 10 µM, *p* ≤ 0.001), *Nestin* (1.00 ± 0.0 v. 0.69 ± 0.11, control v. AZD1208 10 µM, *p* ≤ 0.001), and *OCT4* (1.00 ± 0.0 v. 0.39 ± 0.39, control v. AZD1208 10 µM, *p* ≤ 0.001). Similar results were seen following treatment with 20 µM concentration of AZD1208. (**B**) Immunoblotting demonstrated a decrease in protein expression of stemness markers Nestin, Oct4, and Nanog with increase concentrations of AZD1208 (0–20 µM). Vinculin served as a loading control. Data are reported as mean ± SEM and represent at least three biologic replicates. * *p* ≤ 0.05, *** *p* ≤ 0.001.

Prior studies have demonstrated that PIM kinases support the stem cell-like cancer cell (SCLCC) phenotype in primary hepatoblastoma which may play an important role in the progression of metastatic disease [10]. We noted that the metastatic hepatoblastoma cell line, HLM_2, had higher levels of mRNA abundance of stemness markers *Oct4*, *Nanog*, *Nestin*, and *Sox2* compared to the parent cell line, HuH6 [9], so we sought to explore the effects of PIM kinase inhibition on metastatic hepatoblastoma stemness. Using qPCR, we evaluated the change in mRNA abundance of known stemness markers *Nanog*, *Sox2*, *Nestin*, and *Oct4.* Treatment with AZD1208 (10 µM) decreased the expression of each of the markers; *Nanog* (1.00 ± 0.0 v. 0.45 ± 0.47, untreated v. AZD1208, *p* ≤ 0.05), *Sox2* (1.00 ± 0.0 v. 0.25 ± 0.34, untreated v. AZD1208, *p* ≤ 0.001), *Nestin* (1.00 ± 0.0 v. 0.69 ± 0.11, untreated v. AZD1208, *p* ≤ 0.001), and *Oct4* (1.00 ± 0.0 v. 0.39 ± 0.39, untreated v. AZD1208, *p* ≤ 0.01), (Figure 2A). Similar results were seen with treatment with increased concentration of AZD1208 (20 µM), with a decreased expression of *Nanog*, *Sox2*, *Nestin*, and *Oct4*, (Figure 2A). After demonstrating knockdown at the mRNA level, we then used immunoblotting to evaluate changes at the protein level. Immunoblotting demonstrated a decrease in protein expression of stemness markers Nestin, Oct4, and Nanog with increase concentrations of AZD1208 (0–20 µM). Vinculin served as a loading control (Figure 2B).

### 2.4. PIM Kinase Inhibition Did Not Alter Metastatic Hepatoblastoma Cell Motility or Invasion

In primary hepatoblastoma, we have found PIM kinase inhibition resulted in decreased migration and invasion [8]. Additionally, we have found the HLM_2 cells to be more metastatic than primary hepatoblastoma cells [9] so we sought to investigate the effects of PIM inhibition on HLM_2 cell migration and invasion. Our investigations demonstrated no significant difference in HLM_2 cell migration after treatment with 20 µM AZD1208 (cell count 3146 ± 1679 v. 3560 ± 1204, *p* = 0.17) (Supplemental Figure S1C). Similarly, there was not a decrease in HLM_2 invasion after treatment with 20 µM AZD1208 (cell count 3167 ± 642 v. 3461 ± 494, *p* = 0.07) (Supplemental Figure S1D).

### 2.5. HLM_2 Cells Developed Chemotherapeutic Resistance

**Figure 3:**
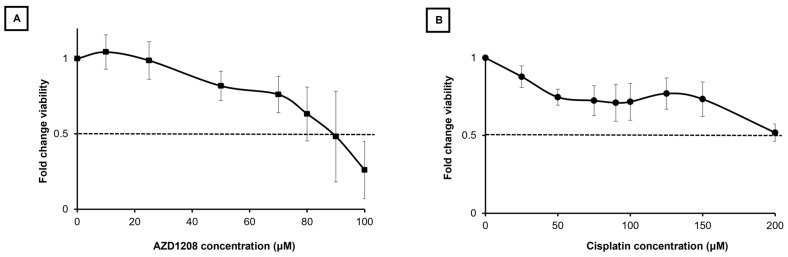
The HLM_2 metastatic hepatoblastoma cell line has chemotherapeutic resistance. (**A**) HLM_2 cells (1.5 × 10^3^ per well) were plated in 96-well plates and treated with increasing concentrations of AZD1208 (0–100 μM) for 72 h. Viability was assessed with almarBlue assay. AZD1208 decreased viability with a calculated lethal dose 50% (LD_50_) of 90.5 μM. (**B**) HLM_2 cells (1.5 × 10^3^ per well) were plated in 96-well plates and treated with increasing concentrations of cisplatin (0–200 μM) for 72 h. Viability was assessed with almarBlue assay. The HLM_2 cells were resistant to cisplatin, with a calculated LD_50_ of 233 μM. Data are reported as mean ± SEM and represent at least three biologic replicates. Dashed line represents concentration associated with LD_50_.

We next explored the effects of PIM kinase inhibition with AZD1208 and the commonly used therapeutic agent, cisplatin, on HLM_2 cell viability. Treatment with increasing concentrations of AZD1208 (0–100 µM) decreased HLM_2 cell viability with a calculated lethal dose 50% (LD_50_) of 90.5 μM (Figure 3A) which is relatively high. We found the HLM_2 cells to similarly be resistant to cisplatin, with a LD_50_ of 233 μM (Figure 3B).

### 2.6. Combination Therapy of AZD1208 and Cisplatin Resulted in Synergy

**Figure 4:**
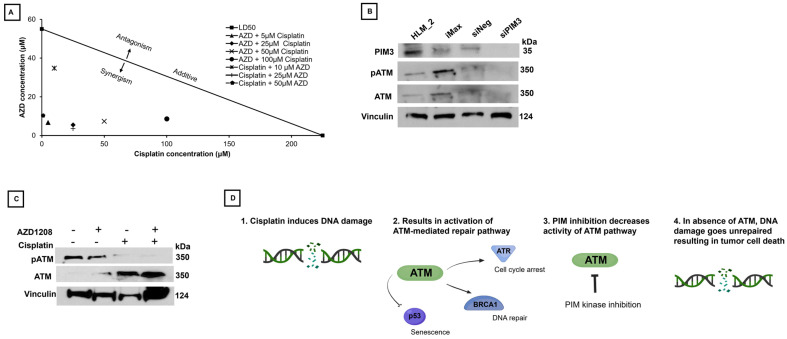
Treatment of HLM_2 cells with AZD1208 and cisplatin is synergistic. (**A**) HLM_2 cells (1.5 × 10^3^ per well) were plated in 96-well plates and treated with combinations of AZD1208 and cisplatin at concentrations below the LD_50_ of AZD1208 (0–60 µM) and cisplatin (0–200 µM). Isobolograms were constructed, and combination indices (CIs) determined. Each combination of therapy resulted in synergy (CI < 1) between the two agents. (**B**) Immunoblotting demonstrated a decrease in pATM, a modulator of DNA repair, after knockdown of PIM3, suggesting a mechanism for the observed synergy. (**C**) Immunoblotting showed that cisplatin increased total ATM expression. Treatment with pan-PIM inhibitor, AZD1208 (50 µM), resulted in decreased pATM and prevented the phosphorylation of the cisplatin-induced increase in total ATM. (**D**) The proposed mechanism of synergy between PIM inhibition and cisplatin is depicted in the cartoon. Cisplatin induces DNA damage which some tumor cells may evade by activating ATM and its downstream targets. PIM kinase inhibition decreases phosphorylated ATM, the active form, allowing for cisplatin-induced DNA damage to commence. This pathway plays a critical role in cell cycle arrest, DNA repair, and senescence. Created with biorender.com [13].

As PIM kinases have been shown to be modulators of chemotherapeutic resistance in malignancy, we evaluated the potential for synergy between AZD1208 and cisplatin. We treated HLM_2 cells using a combination of doses of AZD1208 and cisplatin based on the 72 h viability data (Figure 3). An isobologram was constructed (Figure 4A) which demonstrated that combinatorial therapy resulted in a decrease in the LD_50_ of either agent with combination indices (CIs) less than 1, indicating synergy [12] (Figure 4A).

Immunoblotting was utilized to evaluate mechanisms for the synergy between AZD1208 and cisplatin. We initially investigated the effects of PIM3 knockdown using siRNA. After confirmation of PIM3 knockdown (Figure 4B), we found a decrease in phospho-ATM (p-ATM), the active form of ATM protein (Figure 4B), with little change in total ATM protein expression (Figure 4B). We next evaluated these effects utilizing cisplatin and AZD1208. Treatment with cisplatin increased total ATM expression (Figure 4C). There was a decrease in ATM phosphorylation with AZD1208 treatment (Figure 4C) and AZD1208 prevented phosphorylation of the cisplatin-induced increase in total ATM.

## 3. Discussion

One of the major obstacles in advancing the study of metastatic hepatoblastoma is the lack of models for investigation. Studies in other malignancies including melanoma, osteosarcoma, and breast and prostate cancers have similarly found the metastatic models to be biologically different than the primary tumors [14], further underscoring the importance of metastatic models in the quest for new methods of treating metastatic disease. Similar to our findings in generating HLM_2 cells, Ruibin et al. generated a metastatic ovarian cancer cell line which had higher expression of stemness markers and found these cells to be more proliferative and chemoresistant than the parent line [15]. To our knowledge, HLM_2 is the only metastatic hepatoblastoma cell line available, and these investigations are the first to explore potential therapeutics in directly treating this type of metastatic hepatoblastoma model.

Chemotherapeutic resistance remains a major impediment in the treatment of all cancers, but especially hepatoblastoma. There are multiple mechanisms by which hepatoblastoma cells evade chemotherapies including alterations of drug uptake, increased drug efflux or metabolism, as well as modulating DNA repair, apoptosis, the tumor microenvironment and epithelial-mesenchymal transition [16]. One enzyme implicated in chemoresistance is ATM, a member of the phosphatidylinositol 3-kinase-related kinase family, which functions in DNA damage response and cell cycle checkpoint regulation [17]. Cisplatin functions as a chemotherapeutic agent by inducing DNA damage in cancer cells. This DNA damage signal brings inactive ATM dimers to the breaks in DNA where they are converted to active monomers via phosphorylation. These active ATM monomers coordinate the DNA repair process [18] allowing tumor cells to evade cisplatin-induced DNA damage.

PIM kinases have been implicated in chemoresistance in numerous cancer types via a variety of mechanisms. In the current study, we showed that PIM3 knockdown or inhibition resulted in decreased phosphorylation of ATM and decreased cell viability with cisplatin treatment. Similar findings have been reported in the cancer literature. Hsu et al. demonstrated in prostate cancer cells that paclitaxel-induced DNA damage was increased after siRNA PIM1 knockdown [19]. This increase in DNA damage was associated with an increase in pATM [19]. Zirkin et al. found that in PIM2 overexpressing sarcoma cells exposed to irradiation had higher levels of pATM and reduced amounts of DNA strand breaks compared to empty vector controls [20]. In lymphoma cells, it has been suggested that PIM2 expression is induced by ATM activation after detection of DNA strand breaks [21]. Chen et al. found that after exposure to irradiation, PIM3 silenced pancreatic carcinoma cells had increased evidence of DNA damage and lower levels of pATM compared to the PIM3 overexpressing cells [22].

The translatability of the current findings is supported by recently completed and ongoing clinical trials with these agents. There are several ATM inhibitors that are undergoing Phase I clinical trials for locally advanced tumors: M4076 (NCT04882917), XRD-0394 (NCT05002140), AZD0156 (NCT02588105), or for brain cancer in combination with radiation therapy: AZD1390 (NCT03423628) and WSD0628 (NCT04917145); only one ATM inhibitor has progressed to Phase II trial, ART0380 (NCT05798611) [23]. None of these studies are in pediatric patients. Clinical trials with PIM kinase inhibitors have demonstrated a positive safety profile and efficacy in several tumor types and these inhibitors have advanced to Phase IV testing [23]. Importantly, there are Phase I studies recruiting to study the effects of PIM kinase inhibitors in pediatric malignancies (NCT04238819 and NCT02644460) [23].

The current study is not without limitations. The use of a tail vein model of metastases to generate the HLM_2 cell line comes with the disadvantage of allowing the cells to bypass the initial steps of the metastatic cascade [9]. However, the ability to more quickly generate metastatic disease, compared to the development of spontaneous metastasis, does hasten the pace at which experiments may be conducted. Future investigations will explore other mechanisms of PIM kinase regulation of chemoresistance in metastatic hepatoblastoma such as regulation of modulation of drug influx and efflux pumps and anti-apoptotic activity as well as the role of PIM kinase in altering resistance of other chemotherapeutics used for metastatic hepatoblastoma.

In summary, we have conducted the first experiments of a metastatic hepatoblastoma cell line to explore the potential of small molecule inhibition of PIM kinases as a therapeutic strategy. Our findings suggest that PIM kinases regulate proliferation through alterations in the cell cycle of metastatic cells, potentially through p21. Additionally, metastatic cells showed increased chemoresistance compared to their primary cell line, consistent with findings from other studies. This chemoresistance was overcome by synergistic treatment with PIM inhibition, likely through regulation of the ATM DNA damage repair pathway. Altogether, these findings support the need for further investigations into PIM kinase inhibition as a therapeutic strategy in metastatic hepatoblastoma.

## 4. Methods

### 4.1. Cells and Cell Culture

We utilized the metastatic human hepatoblastoma cell line, HLM_2, which was previously established through a series of tail vein injections to create pulmonary metastases from the human long-term passaged hepatoblastoma cell line, HuH6 [9]. Briefly, HuH6^Luc^ cells were generously provided by the Hjelmeland laboratory (Anita Hjelmeland, University of Alabama at Birmingham (UAB), Birmingham, AL, USA) and were established by stable transfection of HuH6 cells with the luciferase reporter cloned into the pCDH-CMV-MCS-EF1a-Puro lentiviral vector (System Biosciences, Palo Alto, CA, USA). Metastatic hepatoblastoma cells were established through a series of tail vein injections of HuH6^Luc^ and dissociation of the subsequent pulmonary metastases. The cells were maintained in culture in 37 °C and 5% CO_2_ in Dulbecco’s Modified Eagle’s Medium supplemented with 10% fetal bovine serum (HyClone, GE Healthcare Life Sciences, Logan, UT, USA), 1 µg/mL penicillin/streptomycin (Gibco, Carlsbad, CA, USA), and 2 mmol/L L-glutamine (Thermo Fisher Scientific, Waltham, MA, USA). Cells were tested and deemed free of Mycoplasma infection by the Universal Mycoplasma Detection Kit (30-1012K, ATCC). Cell lines were verified within the last 12 months using short tandem repeat analysis Genomics Core, University of Alabama at Birmingham (UAB), Birmingham, AL, USA.

### 4.2. Antibodies and Reagents

Primary antibodies included rabbit monoclonal anti-PIM1 (3247S), anti-PIM2 (4730), anti-PIM3 (4165), anti-p21 (2947S), anti-ATM (2873S), anti-phospho-ATM (4526S), nanog (3580S), nestin (73349S), and anti-vinculin (13901S) from Cell Signaling Technology (Beverly, MA, USA), rabbit polyclonal anti-phospho-p21 (ab47300), Oct4 (19857) from Abcam (Cambridge, MA, USA), and mouse monoclonal anti-β-actin (A1978) from Sigma Aldrich (St. Louis, MO, USA). AZD1208, a pan-PIM kinase inhibitor, and cisplatin were purchased from Selleckchem (Houston, TX, USA).

### 4.3. Transient Knockdown of PIM3 with siRNA

HLM_2 cells were transfected with either PIM3 or control small interfering RNAs (siRNAs) at 40 nM concentration with Lipofectamine RNAiMax (Thermo Fisher Scientific) according to the manufacturer’s protocol [10]. PIM3 siRNA (ON-TARGETplus SMARTpool and control siRNA (siNeg, ON-TARGETplus Non-targeting Pool) were obtained from Dharmacon (Dharmacon, GE Life Sciences, Lafayette, CO, USA).

### 4.4. Cell Proliferation

To evaluate proliferation, HLM_2 cells (5 × 10^4^ cells per well) were plated in 12-well plates after transfection with siRNA for 24 h as described above. Cells were incubated for 24, 48, or 72 h, trypsinized, stained with trypan blue (0.4%, Gibco), and counted with a hemocytometer at each time point. Similarly, to evaluate the effects of pan-PIM kinase inhibition on HLM_2 cell proliferation, HLM_2 cells (5 × 10^4^ cells per well) were plated in 12-well plates. They were allowed to adhere, and the treatment group received 20 µM AZD1208. Cells were incubated for 24, 48, or 72 h, trypsinized, stained with trypan blue (0.4%, Gibco), and counted with a hemocytometer at each time point.

### 4.5. Cell Cycle

HLM_2 cells (1 × 10^6^) were plated in low serum (4% FBS) media, allowed to attach, and incubated for 24 h. Cells were trypsinized, washed with PBS, and fixed in cold 100% ethanol. Cells were stained with 20 μg/mL propidium iodine (Invitrogen, Thermo Fisher, Eugene, OR, USA) and 0.2 mg/mL RNAse A (Invitrogen) in 0.1% Triton X (Active Motif, Carlsbad, CA, USA). The Attune NxT Flow Cytometer (Invitrogen) was used to obtain data and analysis conducted with FlowJo software (FlowJo, LLC, Ashland, OR, USA).

### 4.6. Immunoblotting

Radio-immunoprecipitation assay (RIPA) buffer supplemented with protease inhibitors (Sigma Aldrich), phosphatase inhibitors (Sigma Aldrich), and phenyl-methane-sulfonyl-fluoride (Sigma Aldrich) was used to lyse cells. Immunoblotting, gel transfer, and immunodetection were performed as previously described [8]. The Precision Plus Protein Kaleidoscope molecular weight marker (Bio-Rad, Hercules, CA, USA) confirmed the expected size of target proteins. β-actin or vinculin expression served as an internal control to confirm equal protein loading.

### 4.7. Quantitative Real-Time PCR

The iScript cDNA Synthesis kit (Bio-Rad) was used to synthesize cDNA in a 20 µL reaction mixture with 1 µg of RNA. For quantitative real-time PCR (qPCR), SsoAdvanced SYBR Green Supermix (Bio-Rad) was utilized according to manufacturer’s protocol. Probes for *NESTIN*, octamer-binding transcription factor 4 (*OCT4*), homeobox protein *NANOG*, sex determining region Y-box 2 (*SOX2*), and *β-ACTIN* were obtained (Applied Biosystems, Foster City, CA, USA) and checked for non-specific binding using the basic local alignment as previously described [9,24] qPCR was performed with 10 ng cDNA in 20 μL reaction volume. An Applied Biosystems 7900HT cycler (Applied Biosystems) performed amplification under the following cycling conditions: 95 °C for 2 min, 39-cycle amplification at 95 °C for 5 s, and 60 °C for 30 s. β-actin was utilized as an internal control. Gene expression was calculated using the ΔΔCt method [25] and reported as mean fold change ± SEM.

### 4.8. Cell Viability and Treatment Synergy

The alamarBlue Cell Viability Assay (Thermo Fisher Scientific) was used to measure cell viability. HLM_2 cells (1.5 × 10^6^ per well) were plated in 96-well plates and treated with increasing concentrations of AZD1208 (0–125 µM) or cisplatin (0–200 µM). Following 72 h of treatment, 10 µL of alamarBlue reagent was added to each well and the absorbance was measured at excitation wavelength of 562 nm and emission wavelength of 595 nm using a microplate reader (BioTek Gen5, BioTek, Winooski, VT, USA). For combination studies, HLM_2 cells were treated with either AZD1208 alone, cisplatin alone, or a combination of AZD1208 and cisplatin at varying concentrations for 72 h. The median lethal dose (LD_50_) of each drug and their combinations was determined. Isobolograms were constructed and combination indices (CI) were calculated using the method of Chou and Talalay with CI values less than 1 indicating synergy, equal to 1 indicating an additive effect, and more than 1 indicating antagonism [12].

### 4.9. Migration and Invasion

Migration assays were conducted as previously described [9]. Briefly, 8 µm micropore Transwell inserts from 24-well culture plates (Corning Life Sciences, Corning, NY, USA) were coated with collagen I (10 µg/mL, MP Biomedicals, Santa Ana, CA, USA) for 4 h at 37 °C then washed with PBS. For invasion assays, the inside of the inserts were coated with 50 µL of Matrigel (1 mg/mL, BD Biosciences, San Jose, CA, USA) for 4 h at 37 °C. HLM_2 cells were plated in 6 well plates, allowed to adhere, treated with AZD1208 (0 or 20 µM) for 24 h and 3 × 10^4^ cells were placed in each insert with 350 µL of the respective conditioned media in the bottom of the insert. Cells were allowed to migrate through the membrane or invade through the Matrigel for 24 h. The inserts were fixed with 3% paraformaldehyde and stained with 1% crystal violet. A light microscope obtained images of the inserts and the number of cells in seven random fields per insert were counted using ImageJ (National Institutes of Health (Bethesda, MD, USA) and the Laboratory for Optical and Computational Instrumentation (Madison, WI, USA) (https://imagej.nih.gov/ij, accessed on 1 June 2023).

### 4.10. Data Analysis

All experiments were repeated with at least three biologic replicates and data reported as mean ± standard error of the mean (SEM). To determine statistical significance, a Student’s *t*-test or ANOVA was used, with *p* ≤ 0.05 considered statistically significant.

## Data Availability

There were no data sets generated for the current studies.

## Supplementary Materials

The following supporting information can be downloaded at: https://www.mdpi.com/article/10.3390/ijms25010427/s1.
