# Supplementary material for: PIM Kinase Inhibition Attenuates the Malignant Progression of Metastatic Hepatoblastoma
Source: Int J Mol Sci. 2023 Dec 28;25(1):427. doi: 10.3390/ijms25010427 (PMC10778668; doi:10.3390/ijms25010427)
Supplement: Supplementary file 1 [file ijms-25-00427-s001.zip › ijms-2722075-supplementary.docx]

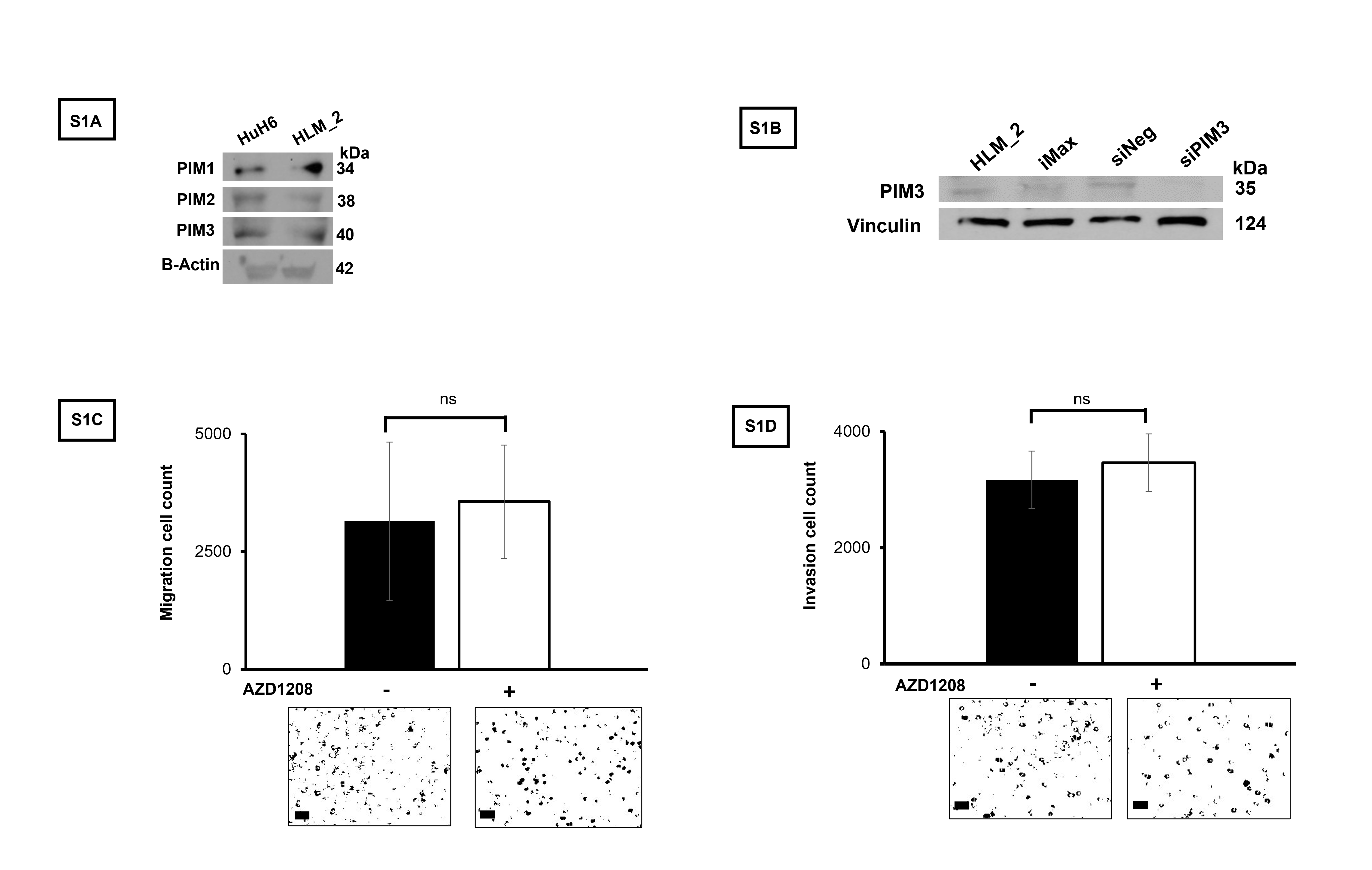


**Figure S1. HLM_2 metastatic hepatoblastoma cells express PIM kinases and migration and invasion are not affected by PIM inhibition.** (**S1A**) Protein from the parent primary hepatoblastoma cell line, HuH6, is provided in the first lane. HLM_2 cells express PIM kinases. (**S1B**) siRNA was utilized to knockdown the expression of PIM3 in HLM_2 cells, vinculin served as a loading control. (**S1C, D**) Preliminary studies of migration and invasion assays were conducted using 8 µm micropore Transwell inserts from 24-well culture plates coated with collagen type I. For invasion assays, the inside of the inserts were coated with 50 µL of Matrigel. HLM_2 cells were plated in 6 well plates, allowed to adhere, treated with AZD1208 (0 or 20 µM) for 24 h and 3 × 10^4^ cells were placed in each insert with 350 µL of the respective conditioned media in the bottom of the insert. Cells were allowed to migrate through the membrane or invade through the Matrigel for 24 hours. (**S1C**) There was no significant difference in HLM_2 cell migration after treatment with 20 µM AZD1208 (cell count 3146 ± 1679 v. 3560 ± 1204, *p* = 0.17). (**S1D**) There was not a decrease in HLM_2 invasion after treatment with 20 µM AZD1208 (cell count 3167 ± 642 v. 3461 ± 494, *p* = 0.07). Representative photomicrographs are pictured; scale bar insets represent 100 µM. Data represent two biologic replicates and are reported as mean ± SEM. ns=not significant.
